# Vitamin D Status in Roma Mothers and Newborns: Socioeconomic Factors and Impact on Neonatal Outcome

**DOI:** 10.3390/nu16244361

**Published:** 2024-12-18

**Authors:** Andreea Bianca Stoica, Maria Oana Săsăran, Laura Mihaela Suciu, Adina Huțanu, Claudiu Mărginean

**Affiliations:** 1 Doctoral School of Medicine, ‘George Emil Palade’ University of Medicine, Pharmacy, Sciences and Technology of Târgu Mureș, Gheorghe Marinescu Street No. 38, 540136 Târgu Mureș, Romania; andreeabstoica9@gmail.com; 2 Department of Pediatrics 3, ‘George Emil Palade’ University of Medicine, Pharmacy, Sciences and Technology of Târgu Mureș, Gheorghe Marinescu Street No. 38, 540136 Târgu Mureș, Romania; 3 Department of Pediatrics 4, ‘George Emil Palade’ University of Medicine, Pharmacy, Sciences and Technology of Târgu Mureș, Gheorghe Marinescu Street No. 38, 540136 Târgu Mureș, Romania; lauramihaelasuciu@yahoo.com; 4 Department of Laboratory Medicine, ‘George Emil Palade’ University of Medicine, Pharmacy, Sciences and Technology of Târgu Mureș, Gheorghe Marinescu Street No. 38, 540136 Târgu Mureș, Romania; adina_hutanu03@yahoo.com; 5 Center for Advanced Medical and Pharmaceutical Research, ‘George Emil Palade’ University of Medicine, Pharmacy, Sciences and Technology of Târgu Mureș, Gheorghe Marinescu Street No. 38, 540136 Târgu Mureș, Romania; 6 Department of Obstetrics and Gynecology 2, ‘George Emil Palade’ University of Medicine, Pharmacy, Sciences and Technology of Târgu Mureș, Gheorghe Marinescu Street No. 38, 540136 Târgu Mureș, Romania; marginean.claudiu@gmail.com

**Keywords:** vitamin D deficiency, Roma population, newborns, maternal health, socioeconomic factors

## Abstract

Background: The Roma are a socioeconomically disadvantaged, marginalized community with reduced access to education, social services, and healthcare. Despite the known health risks they are exposed to, we have limited data about a wide range of health outcomes in this population, including vitamin D deficiency. The aim of this study was to investigate prevalence of vitamin D deficiency and its impact on the anthropometric outcomes of newborns in a group of Roma mothers and their infants in Romania. Methods: In total, 131 Roma women and 131 newborns were included in the study. Vitamin D levels in both mothers and newborns, as well as the birth weight, length, and head circumference of newborns, were recorded at birth. We also assessed socioeconomic factors, including education, employment status, income, and living conditions, as well as factors that influence vitamin D status, including sun exposure, use of sunscreen, fish consumption, and skin type. Results: All mothers and almost all newborns had vitamin D insufficiency or deficiency, with 25-hydroxivitamin D levels below 30 ng/mL. Maternal vitamin D status was significantly correlated with neonatal vitamin D levels (*p* < 0.01) but not with anthropometric outcomes such as birth weight (*p* = 0.57), birth length (*p* = 0.53), or head circumference (*p* = 0.96). Most study participants had a low socioeconomic status, reporting severe deficiencies in education, employment status, household income, and living conditions. Conclusions: Vitamin D deficiency is a significant public health issue among Roma women and their newborns, which may be compounded by the socioeconomic challenges of this vulnerable population.

## 1. Introduction

Vitamin D is vital for both maternal and neonatal health, having a crucial role in calcium absorption, bone development, and immune function [1]. During pregnancy, adequate levels of vitamin D are essential for fetal growth and the prevention of complications such as preeclampsia and gestational diabetes. For newborns, sufficient vitamin D levels help prevent rickets, poor bone formation, and immune system deficiencies [2]. In recent years, research has focused on the relationship between maternal and neonatal vitamin D levels, as well as between maternal vitamin D deficiency and infant health outcomes [3]. Studies have shown a correlation between neonatal vitamin D levels at birth and maternal vitamin D plasma levels [4], and vitamin D supplementation is recommended for pregnant women to prevent adverse neonatal health outcomes [3]. Vitamin D levels also seem to influence the neuropsychiatric development of the offspring. A systematic review has showed that whilst maternal vitamin D deficiency does not associate with autism spectrum disorders, it can associate with attention deficit/hyperactivity disorders and schizophrenia [5]. Hence, research on the relationship between maternal and offspring vitamin D level remains a promising topic of investigation.

Vitamin D deficiency remains a significant global health issue, especially in socioeconomically disadvantaged populations [6]. One such population is the Roma, the largest ethnic minority in Europe, with a significant presence in Romania. The Roma have long been marginalized, and many live in conditions of extreme poverty, with limited access to healthcare, proper nutrition, and social services [7]. Due to socioeconomic constraints, Roma women face multiple barriers to adequate prenatal care, preventing them from accessing health services that provide dietary guidance or vitamin supplementation during pregnancy [8]. Additionally, Roma families often have diets low in vitamin D-rich foods, live in overcrowded conditions, and spend limited time outdoors [9]. These conditions increase the risk of vitamin D deficiency both for Roma mothers and their newborns, whose vitamin D status depends directly on that of the mother [10].

Despite the known health risks, there is a lack of research on vitamin D deficiency in the Roma population. This study aims to address this gap by investigating the prevalence of vitamin D deficiency and its impact on the anthropometric outcomes of newborns in a group of Roma mothers and their infants in Romania.

## 2. Materials and Methods

### 2.1. Study Population

Pregnant Roma women in their last trimester of pregnancy, between gestational weeks 37 and 42, were conveniently recruited between February 2023 and September 2023 when presenting for delivery to the Obstetrics Clinic of the County Clinical Hospital of Târgu Mureș, Romania.

In total, 216 mothers were approached for the study. The inclusion criteria were the following: Roma ethnicity, term birth (between gestational weeks 37 and 42), singleton pregnancy, and willingness to participate in the study. Premature newborns, newborns from twin pregnancies, mothers with chronic diseases and/or pregnancy-associated conditions, such as diabetes, untreated thyroid disorders (hypothyroidism or hyperthyroidism), renal insufficiency, arterial hypertension, autoimmune diseases, and hematological disorders, as well as those who did not sign the informed consent form, were excluded from the study. After applying the inclusion and exclusion criteria, 131 mothers and their newborns were included in the study.

### 2.2. Data Collection

Data on maternal characteristics included age, urban/rural background, civil status, education level, employment status, household income, and health insurance coverage. Living conditions were assessed based on the type of residence, heating system, and availability of running water. Additional information was gathered regarding sun exposure (the number of days spent outdoors between 10 a.m. and 4 p.m. per week over the past 6 months), use of sunscreen, fish consumption, and the number of medical visits during pregnancy. Parity and the mode of delivery (vaginal or cesarean) were also recorded. Skin type was determined using the Fitzpatrick classification.

Newborn characteristics included gestational age, sex, birth weight, birth weight classification according to gestational age, length, head circumference, APGAR score, and type of feeding (breastfeeding, formula, or mixed).

### 2.3. Vitamin D Quantification

We collected a 10 mL venous blood sample from the mother at admission and a 5 mL sample from the umbilical cord of the newborn at birth. The blood was collected into biochemistry tubes using the Vacutainer system. After collection, the samples were allowed to coagulate at room temperature for 15 min, then centrifuged at 3000 rpm for 2 min to separate the serum. Following centrifugation, the serum was frozen at −20 °C for further analysis.

The quantification of vitamin D in the laboratory was performed using the electrochemiluminescence technique (ECLIA) on a Cobas e402 analyzer with dedicated reagents, internal controls, and calibrators (Roche Diagnostics GmbH, Mannheim, Germany). The process is based on a competitive principle, wherein a ruthenium-labeled vitamin D binding protein (VDBP) functions as a capture protein for endogenous vitamin 25-hydroxyvitamin D_3_ (25-(OH)_3_ D) and 25-hydroxyvitamin D_2_ (25-(OH)_2_ D). The procedure involves several steps. First, the patient sample is pretreated with a reagent that facilitates the release of 25-(OH)_2_ D and 25-(OH)_3_ D from the endogenous VDBP. Once released, the free vitamin 25-(OH)_2_ D and 25-(OH)_3_ D bind to the ruthenium-labeled VDBP, forming a stable complex. A specific monoclonal antibody is used to reduce potential interference from the (24,25-(OH)_2_ D) metabolite. In the next step, competition occurs between the ruthenium-labeled VDBP and biotinylated vitamin D. This is achieved by adding biotin-labeled (25-(OH)_2_ D) and streptavidin-coated microparticles to the reaction medium. The concentration of vitamin D in the samples is then determined by extrapolating results from a pre-established calibration curve, where signal intensity is inversely proportional to the vitamin D concentration. Accuracy was verified using two controls with traceable values, showing relative deviations of less than ±10%, with a relative deviation of 0.52%. Precision was determined according to the NCCLS protocol, with a within-run CV of 5.71%. The analyzer assay is designed to have a precision < 10%.

Vitamin D insufficiency was defined as serum 25-(OH)_3_ D levels between 20 and 29.9 ng/mL, whereas levels of 19.9 ng/mL or lower were classified as deficiency [11].

### 2.4. Ethical Statements

Each mother signed the informed consent for herself and her newborn included in the study.

The study was conducted in accordance with the principles stated in the Declaration of Helsinki. The research protocol was approved by the ethics committees of Mureș County Hospital (approval no. 437; approval date: 9 January 2023) and the ‘George Emil Palade’ University of Medicine, Pharmacy, Science, and Technology of Târgu Mureș (approval no. 3315; approval date: 19 July 2024).

### 2.5. Statistical Analysis

Data analysis was performed using GraphPad Prism v.9.0.2 (GraphPad Software, Boston, MA, USA). Descriptive statistics included mean, standard deviation (SD), median, and percentage. The distribution of quantitative variables was assessed using the Kolmogorov–Smirnov normality test. For comparisons of means, Student’s *t*-test was used for normally distributed data, while the Mann–Whitney test was applied for non-normally distributed data. Binary data were analyzed using the chi-squared test. To explore potential correlations between variables, we used linear regression or Pearson–Spearman correlations as appropriate. Statistical significance was set at an α of 0.05, corresponding to a 95% confidence interval (CI).

## 3. Results

The mothers had a mean age of 23 ± 7 years and a mean vitamin D level of 17.84 ± 3.98 ng/mL. The anamnesis revealed that none of the mothers included in the study had taken vitamin D supplements. Vitamin D insufficiency was found in 34 cases (26%) and deficiency in 97 cases (74%). The descriptive characteristics of the mothers included in the study are presented in Table 1.

The data provided by study participants regarding the factors that influence socioeconomic status were concerning: 92% were unemployed, 93% did not finish high school, 89% had a monthly household income of less than 1000 RON (approx. 200 euros), 45% had no health insurance coverage, 53% used wood for heating, and 53% had no running water in their house.

The newborns had a mean birth weight of 3186 ± 467 g and a mean vitamin D level of 19.07 ± 5.38 ng/mL. Vitamin D deficiency was found in 45 cases (34%) and insufficiency in 84 cases (64%). Birth weight was classified as appropriate for gestational age in 79% of newborns, and the mean APGAR score was above 9 both at 1 and 5 min (Table 2).

A gender-based comparison of vitamin D levels was conducted in neonates, as portrayed through Table 3. There were no significant differences in mean vitamin D serum levels between female and male newborns (*p* = 0.97). Furthermore, mean maternal vitamin D levels were similar between the two sexes and the prevalence of vitamin D insufficiency and vitamin D deficiency did not differ significantly in relation to newborn gender (*p* = 0.97).

**Figure 1:**
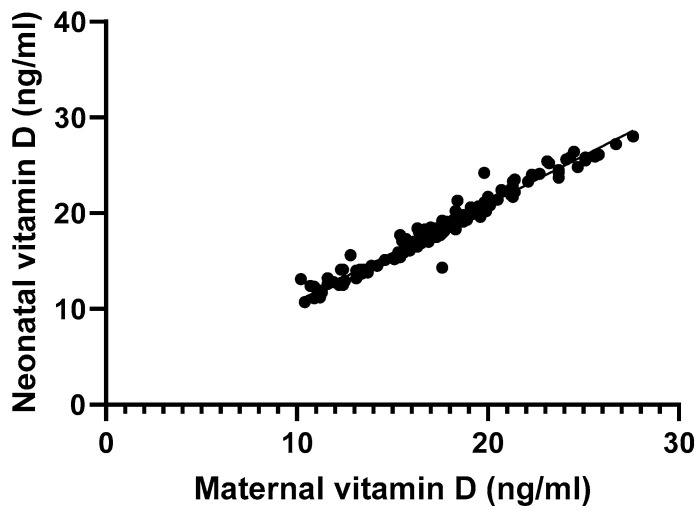
Linear regression correlation between maternal and neonatal serum vitamin D levels.

We sought to establish correlations between maternal and neonatal serum vitamin D levels, as well as neonatal anthropometric parameters. We found a positive, linear correlation between maternal and neonatal serum vitamin D levels (*p* < 0.01, r^2^ = 0.96; Figure 1). However, there was no correlation between maternal vitamin D levels and neonatal birth weight (*p* = 0.57, r^2^ < 0.01), length (*p* = 0.53, r^2^ < 0.01), head circumference (*p* = 0.96, r^2^ < 0.01), APGAR score at 1 min (*p* = 0.61, r^2^ < 0.01), APGAR score at 5 min (*p* = 0.99, r^2^ < 0.01), and newborn weight-for-gestational age classification (*p* = 0.56, r^2^ < 0.01).

**Figure 2:**
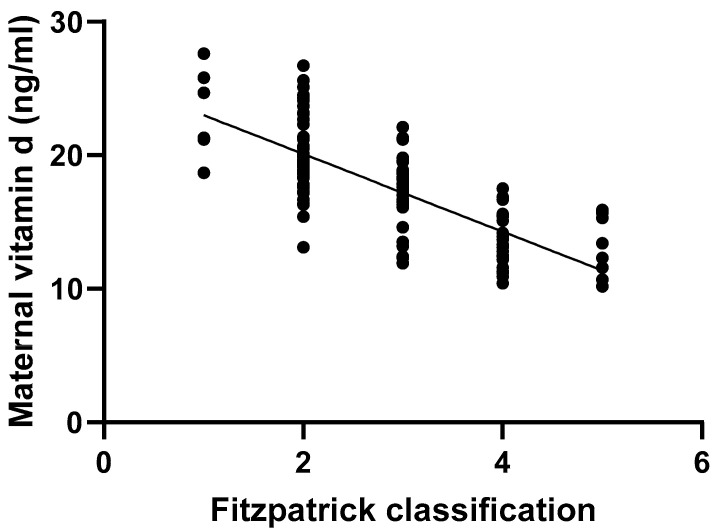
Non-parametric Spearman correlation between maternal serum vitamin D levels and skin type according to the Fitzpatrick classification.

The non-parametric Spearman analysis also yielded a significant correlation in mothers between skin type based on the Fitzpatrick classification and serum vitamin D levels (*p* < 0.01, r = −0.73, 95% CI: −0.80–−0.64). In other words, mothers with the darkest skin pigmentation had the lowest vitamin D levels (Figure 2). Although data regarding the amount of sun exposure varied widely, more than 30% of participants reported spending at least 2 h outdoors each day. However, it is important to note that all of them indicated they were fully clothed during this time.

We found no significant differences in age, background, or type of delivery between mothers with vitamin D insufficiency and vitamin D deficiency. However, there was a significant difference in mean parity, which was higher among mothers with vitamin D deficiency (2.6 ± 1.7 vs. 1.7 ± 1.3; *p* < 0.01). In terms of neonatal variables, we found no significant differences in birth weight, length, head circumference, or APGAR score between newborns belonging to mothers with deficiency vs. vitamin D insufficiency. Chi-squared tests were conducted to assess frequency of various binary data such as newborn weight-for-age division, newborn gender, mode of delivery, and maternal urban/rural background in relation to maternal vitamin D insufficiency or deficiency. However, significantly lower serum vitamin D levels were found in newborns belonging to mothers with vitamin D insufficiency (16.91 ± 2.97 ng/mL vs. 25.26 ± 5.94 ng/mL; *p* < 0.01). We also found a significantly higher percentage of newborns who were large for gestational age in the insufficiency group (3% vs. 1%), but overall, they represented a small percentage of the total number of newborns. Furthermore, we found a significantly higher prevalence of male gender among newborns belonging to mothers with vitamin D deficiency (odds ratio 7.85; *p* < 0.01) (Table 4).

Then, we examined the impact of certain confounders, such as maternal age, parity, and Fitzpatrick skin classification, on maternal vitamin D levels, using multiple linear regression. Each confounder was assessed both individually and within models of combined variables (Table 5). As previously illustrated in Figure 2, the Fitzpatrick classification was the only confounder that has influenced maternal vitamin D levels.

We also analyzed the impact of socioeconomic factors on maternal vitamin D levels (Table 6). We found a significant, positive association between regular fish intake, of at least once per week, and higher maternal vitamin D levels (*p* < 0.01). Although the other individual variables did not associate with maternal vitamin D serum levels, when analyzing a combination of the socioeconomic factors while taking into consideration two-way interactions, urban background and fish intake of at least once per week significantly influenced the outcome variable (*p* < 0.01).

**Figure 3:**
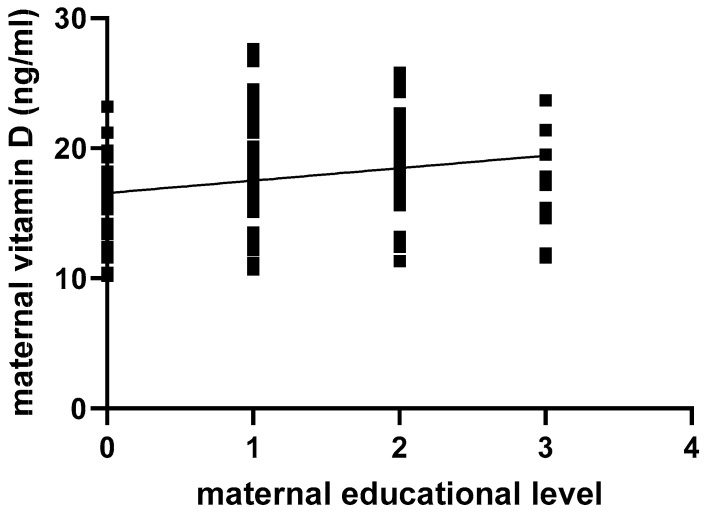
Correlation between maternal education level and maternal vitamin D levels: 0, no school enrollment; 1, primary school; 2, middle school; 3, high school.

**Figure 4:**
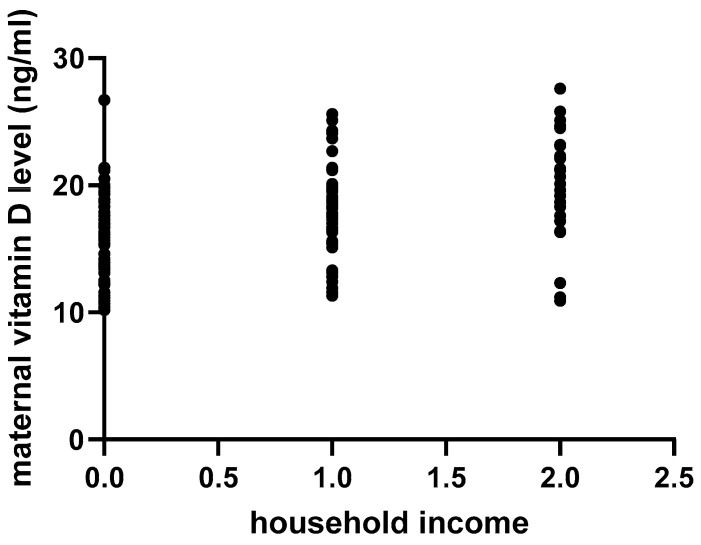
Correlation between household income and maternal vitamin D levels: 0, <500 RON/month; 1, 500–1000 RON/month; 2, >1000 RON/month.

As two other socioeconomic factors (education level and household income), expressed as categorical variables, were divided into multiple categories (as projected in Table 1), we conducted a separate, non-parametric Spearman correlation for each individual variable. A higher educational level was significantly correlated with higher vitamin D levels (*p* = 0.01; r = 0.20; 95% CI 0.03–0.36), as illustrated in Figure 3. In similar fashion, a lower household income was considered a risk factor for lower vitamin D levels (*p* < 0.01; r = 0.36; 95% CI 0.20–0.51; Figure 4).

## 4. Discussion

Our study reveals a high prevalence of vitamin D deficiency in a population of Roma mothers in Romania, highlighting an important public health concern that also extends to their newborns. To put these findings into context, it is important to understand the socioeconomic background of Roma communities and their effect on the health status of this vulnerable population.

The Roma have faced centuries of marginalization and discrimination across Europe, including Romania, resulting in persistent socioeconomic disadvantages with a profound effect on health outcomes, including maternal and child health [12]. Roma communities often experience poverty and social exclusion, with estimates suggesting that in certain European countries up to 80% of Roma live below the national poverty line [12]. This economic marginalization is compounded by high rates of unemployment, substandard housing conditions that lack basic infrastructure such as running water or electricity, and reduced access to social services, including healthcare and education, due to a combination of geographic isolation, discrimination, and bureaucratic barriers [12]. These data are confirmed by our findings, as most of the women included in the study reported severe deficiencies in education, employment status, household income, and living conditions, indicative of a low socioeconomic status.

In most Roma communities, these socioeconomic disadvantages translate to much poorer health compared to the general population [13]. Moreover, educational constraints limit health literacy, severely affecting the ability of Roma individuals to navigate health systems and make informed health decisions [14]. Additionally, they frequently report experiences of discrimination when accessing health services on the basis of their ethnicity, economic status, or language [8], leading to mistrust and avoidance of formal healthcare systems.

Systemic issues are also prevalent when it comes to prenatal care. Roma women face multiple barriers to accessing adequate prenatal care, with a direct impact on their overall health during pregnancy. Studies have found that Roma women are significantly less likely to receive prenatal care compared to their non-Roma peers due to geographic isolation, financial constraints, issues with language and/or communication, cultural beliefs and practices, or the attitudes of healthcare providers towards Roma women [15,16]. This also translates into a lack of awareness regarding the recommended dietary supplements throughout the pregnancy. Without regular prenatal care and nutritional guidance, many Roma women do not receive or adhere to vitamin D supplementation recommendations during pregnancy. These issues were also obvious in our study population, as all of the mothers were vitamin D-deficient and none of them attended prenatal care over the course of their pregnancy, many of them presenting to the hospital solely for delivery. Nevertheless, health education interventions tailored to Roma communities have shown promise in improving the situation, even if they are most likely insufficient to address the scale of the problem. For example, in a study on the effects of a long-term, targeted program on prenatal care for Roma women carried out in rural areas of Romania, Mitrut et al. found that the program has substantially increased the number of pregnant Roma women taking up prenatal care and improved their healthcare-seeking behavior after giving birth [17].

The poor socioeconomic background of Roma communities has a direct impact on the vitamin D status of pregnant women. Studies have shown that socioeconomic factors, such as marital status, education, and income, influence vitamin D status by affecting sunlight exposure, dietary choices, or access to healthcare [18,19]. Economic hardship often results in diets lacking in vitamin D-rich foods such as fatty fish, egg yolks, and fortified products [9]. These issues were confirmed by our study, as very low household incomes correlated negatively with serum maternal vitamin D levels, which were also negatively impacted by lack of/scarce fish intake. Low education levels may correlate with reduced awareness of the importance of vitamin D and strategies to maintain adequate levels. As shown in our study, the lowest vitamin D levels were encountered in those mothers who were not even enrolled to school.

Data on the vitamin D status of Roma populations, and especially pregnant Roma women, are limited. In a meta-analysis involving 308 studies with nearly 8 million participants across 81 countries, Cui et al. (2016) reported that 15.7% of the population had serum 25-(OH)_2_ D levels below 30 nmol/L, while 47.9% had levels under 50 nmol/L [20]. Our findings align with previous studies conducted in the region. For instance, Cashman et al. reported an overall pooled estimate of the prevalence of vitamin D levels below 20 ng/mL of 40% in Europe [21]. In Romania, studies have reported similar vitamin D deficiency rates among the general population. In a study involving 5380 adults aged 25–64 years from Romania, Brinduse et al. found an overall prevalence of vitamin D levels below 20 ng/mL of 24.8% [22]. In another study, involving 8024 Romanian adults, Niculescu et al. found that 55.6% of subjects had vitamin D levels below 20 ng/mL and 86.1% below 30 ng/mL [23]. Bucurica et al. reported vitamin D levels of <20 ng/mL in 28.83% of the 11,182 Romanian patients evaluated in their study [24]. None of these studies have assessed vitamin D status among pregnant women. In our study, all included women had vitamin D deficiency, which suggests that Roma women may be at even greater risk due to the compounded effects of socioeconomic disadvantages mentioned above.

The positive, linear correlation between maternal and neonatal serum vitamin D levels observed in our study is consistent with our current understanding of the link between maternal and neonatal vitamin D status [2], and it emphasizes the importance of addressing maternal deficiency for optimal fetal development. Interestingly, we found no significant correlation between maternal vitamin D levels and the anthropometric measurements of newborns, including birth weight, length, and head circumference. This lack of association contrasts with previous studies that have suggested a link between maternal vitamin D status and fetal growth. For instance, an umbrella review of 16 systematic reviews and meta-analyses on the effects of vitamin D in pregnancy on maternal and offspring health outcomes by Chien et al. found that low vitamin D levels in the offspring were associated with significantly reduced birth weight and low head circumference [3]. Other studies have also reported an association between low maternal vitamin D levels and low birth weight [25,26,27,28]. However, our findings are not entirely unexpected, as the relationship between vitamin D and fetal growth remains controversial and complex. One randomized controlled trial has emphasized the need for implementing screening programs among pregnant women, for optimal introduction of vitamin D supplementation among vitamin D-deficient individuals, which can prevent adverse pregnancy outcomes such as preeclampsia and premature delivery [29]. However, another randomized controlled trial by Roth et al. which enrolled 1164 pregnant women in Bangladesh found that high-dose maternal vitamin D supplementation during pregnancy and the early postpartum period had no significant effect on fetal or infant growth [30]. Other studies, including high-quality randomized controlled trials, have also found that vitamin D status does not influence anthropometric parameters [31,32,33,34]. The inconsistency in findings across studies can be probably attributed to nutritional, environmental, and genetic factors [32], and larger studies with more diverse populations and longitudinal designs are needed to fully elucidate these relationships.

We consider the significantly higher prevalence of male sex among newborns belonging to mothers with vitamin D deficiency compared to those with insufficiency an intriguing finding. While the biological mechanisms are not immediately clear, a recent study suggests a possible link between vitamin D status in mothers, measured during the pre-conceptional period, and offspring sex ratio. In a study involving 1228 women who were trying to obtain a pregnancy, the authors found a positive association between preconception 25-(OH)_2_ D levels and the live birth of a male fetus [35]. This finding is in contrast with the results of our study and may be attributed to the fact that the observed association in the former study was notably stronger among women with elevated levels of high-sensitivity C-reactive protein. Further research is needed to explore the possible interaction between vitamin D status, inflammation, and neonatal outcomes to clarify these conflicting findings.

The socioeconomic disparities outlined above place Roma mothers and their newborns at increased risk for vitamin D deficiency, creating a cycle of health inequity that can persist across generations. Addressing the underlying factors is crucial for developing effective interventions to improve vitamin D status among Roma mothers and their newborns. Future research should explore the long-term health consequences of maternal and neonatal vitamin D deficiency in this population, as well as evaluate the effectiveness of various intervention strategies.

The main strength of our study is that it provides data about vitamin D status and highlights the complex interplay between socioeconomic factors, maternal health, and neonatal outcomes in an under-researched, marginalized community. The most important limitations are the relatively low sample size due to reduced willingness to participate in the study, which may not accurately reflect the vitamin D status of all Roma pregnant women. Another limitation is represented by the study’s cross-sectional design; vitamin D levels were measured before delivery, which might not capture the full picture of vitamin D’s influence throughout gestation. In addition, as with any self-reported data, the accuracy of the information collected is subject to the honesty and memory of participants, which may lead to some level of response bias.

## 5. Conclusions

All mothers and almost all newborns included in this study had vitamin D deficiency, suggesting that this public health issue has a significant burden among Roma mothers and their newborns in Romania, reflecting not only individual health choices but also broader socioeconomic inequities. We found a significant correlation between maternal vitamin D status and neonatal serum vitamin D levels but not between maternal vitamin D status and anthropometric outcomes such as birth weight, length, or head circumference. This study also identified important correlations between socioeconomic factors such as low household income and educational level and maternal vitamin D levels. Further studies are needed to assess the socioeconomic challenges faced by the Roma and their impact on the health status of this vulnerable population.

## Figures and Tables

**Table 1:** Descriptive characteristics of the mothers included in the study.

Variables		Value
Mean age (years)		23 ± 7
Background (%)	Rural	56
	Urban	44
Civil status (%)	Married	58
	Not married	42
Education level (%)	No school enrollment	18
	Primary school	38
	Middle school	37
	High school	7
Employment status (%)	Unemployed (social support)	92
	Employed	8
Household income (%)	<500 RON/month	46
	500–1000 RON/month	43
	>1000 RON/month	11
Health insurance coverage (%)	Yes	55
	No	45
Type of residence (%)	House	100
	Apartment building	0
Heating method (%)	Gas	47
	Wood	53
Running water (%)	Yes	47
	No	53
Fish consumption during pregnancy (minimum once per week) (%)	Yes	18
	No	82
No. of days per week with at least 2 h spent outdoors between 10 a.m. and 4 p.m. in the last 6 months (%)	1	1
	2	16
	3	13
	4	12
	5	19
	6	8
	7	31
Use of sunscreen (%)	Yes	0
	No	100
Skin type according to the Fitzpatrick classification (%)	I	5
	II	44
	III	27
	IV	18
	V	6
Mode of delivery (%)	Vaginal	73
	Cesarean	27
Parity (%)	1	34
	2	29
	3	21
	4	5
	5	5
	6	2
	7	1
	8	2
	10	1
Mean vitamin D level (ng/mL)		17.84 ± 3.98
Vitamin D deficiency (%)	Deficiency (≤19.9 ng/mL)	26
	Insufficiency (20–29.9 ng/mL)	74

**Table 2:** Descriptive characteristics of the newborns included in the study.

Variables		Value
Mean gestational age (weeks)		38.94 ± 1.57
Sex (%)	Male	58
	Female	42
Mean birth weight (g)		3186 ± 467
Birth weight according to gestational age (%)	Appropriate for gestational age	79
	Small for gestational age	18
	Large for gestational age	3
Mean length (cm)		52.25 ± 2.53
Mean head circumference (cm)		33.37 ± 1.42
Mean APGAR score	At 1 min	9.38 ± 0.76
	At 5 min	9.82 ± 0.42
Type of feeding (%)	Breastfeeding	41
	Formula	30
	Mixed	29
Mean vitamin D level (ng/mL)		19.07 ± 5.38
Vitamin D level (%)	Normal	2
	Insufficiency	34
	Deficiency	64

**Table 3:** Comparison of neonatal and maternal vitamin D levels based on newborn gender.

Variable	Male Sex (*n* = 76)	Female Sex (*n* = 55)	*p* Value
Neonatal vitamin D level (ng/mL, mean ± SD)	18.90 ± 4.59	19.31 ± 6.35	0.97
Maternal vitamin D level (ng/mL, mean ± SD)	17.85 ± 4.35	17.82 ± 3.72	0.97
Vitamin D normal level (%)	1	1	0.97
Vitamin D insufficiency (%)	20	14	
Vitamin D deficiency (%)	37	27	

**Table 4:** Comparison between mothers with vitamin D deficiency and vitamin D insufficiency.

Variable		Maternal Vitamin D Insufficiency (*n* = 34)	Maternal Vitamin D Deficiency (*n* = 97)	*p* Value
Neonatal Variables	Birth weight (g)	3209 ± 496.5	3178 ± 459.4	0.74
	Length (cm)	52.21 ± 2.54	52.27 ± 2.53	0.83
	Head circumference (cm)	33.35 ± 1.44	33.41 ± 1.39	0.96
	APGAR score at 1 min	9.3 ± 0.7	9.4 ± 0.8	0.33
	APGAR score at 5 min	9.8 ± 0.4	9.8 ± 0.4	0.78
	Mean serum vitamin D (ng/mL)	25.26 ± 5.94	16.91 ± 2.97	<0.01
Newborn weight-for-age classification	Small for gestational age (%)	4	13	0.03
	Appropriate for gestational age (%)	21	58	
	Large for gestational age (%)	3	1	
Newborn gender	Female (%)	44	14	<0.01
	Male (%)	12	30	
Type of delivery	Vaginal	19	54	0.96
	Cesarian	7	20	
Maternal variables	Mean age (years)	23.36 ± 7.12	23.38 ± 7.50	0.99
	Rural background (%)	14	42	0.84
	Urban background (%)	12	32	
	Mean parity	1.7 ± 1.3	2.6 ± 1.7	<0.01

**Table 5:** Multiple linear regression for the assessment of individual and combined model cofounders (continuous variables) for maternal serum vitamin D levels.

Parameter	Variable	Estimate	Standard Error	95% CI	t Value	*p* Value
β0	Intercept	27.10	4.257	18.67 to 35.52	6.36	<0.01
β1	Maternal age	0.05	0.18	−0.30 to 0.41	0.29	0.76
β2	Maternal parity	−1.18	1.702	−4.55 to 2.18	0.69	0.48
β3	Fitzpatrick classification	−3.71	1.610	−6.90 to −0.53	2.30	0.02
Model 1	Maternal age + parity	<0.01	0.07	−0.13 to 0.14	0.07	0.94
Model 2	Maternal age + Fitzpatrick classification	0.02	0.06	−0.10 to 0.15	0.39	0.69
Model 3	Maternal parity + Fitzpatrick classification	0.52	0.67	−0.80 to 1.86	0.78	0.43
Model 4	Maternal age + parity + Fitzpatrick classification	−0.01	0.02729	−0.07 to 0.03	0.65	0.51

**Table 6:** Multiple linear regression for assessment of individual and combined socioeconomic factors for maternal serum vitamin D levels.

Parameter	Variable	Estimate	Standard Error	95% CI	t Value	*p* Value
β0	Intercept	16.46	0.68	15.11 to 17.81	24.19	<0.01
β1	Background (ref. = rural)	1.502	0.96	−0.40 to 3.41	1.55	0.12
β2	Civil status (ref. = married)	0.55	0.94	−1.32 to 2.43	0.58	0.56
β3	Employment status (ref. = unemployed)	−0.62	2.21	−5.01 to 3.76	0.28	0.77
β4	Fish intake (ref. = no regular intake/week)	6.61	1.31	4.01 to 9.21	5.04	<0.01
Model 1	Background + civil status	−1.50	1.38	−4.24 to 1.22	1.09	0.27
Model 2	Background + employment status	1.41	2.95	−4.43 to 7.26	0.48	0.63
Model 3	Background + fish intake	−4.91	1.92	−8.72 to −1.11	2.55	0.01
Model 4	Civil status + employment status	−0.88	4.32	−9.45 to 7.67	0.20	0.83
Model 5	Civil status + fish intake	0.03	1.97	−3.87 to 3.95	0.02	0.98
Model 6	Employment status + fish intake	−1.45	3.58	−8.55 to 5.65	0.40	0.68

## Data Availability

The data presented in this study are available on request from the corresponding author.
